# Antimicrobial and Genetic Profiles of *Vibrio vulnificus* and *Vibrio parahaemolyticus* Isolated From the Maryland Coastal Bays, United States

**DOI:** 10.3389/fmicb.2021.676249

**Published:** 2021-05-21

**Authors:** Ligia V. da Silva, Sylvia Ossai, Paulinus Chigbu, Salina Parveen

**Affiliations:** ^1^Department of Natural Sciences, CREST Center for the Integrated Study of Coastal Ecosystem Processes and Dynamics in the Mid-Atlantic Region, NOAA Living Marine Resources Cooperative Science Center, University of Maryland Eastern Shore, Princess Anne, MD, United States; ^2^Department of Agriculture, Food and Resource Sciences, Food and Agricultural Sciences Program, University of Maryland Eastern Shore, Princess Anne, MD, United States

**Keywords:** *Vibrio vulnificus*, *Vibrio parahaemolyticus*, serotype, antimicrobial profile, genetic profile

## Abstract

*Vibrio vulnificus and V. parahaemolyticus*, found naturally in marine and estuarine environments, are the leading cause of seafood associated gastrointestinal illness and death. Consumption of improperly cooked crabs and handling of live crabs are potential routes of exposure to pathogenic bacteria such as *V. vulnificus* and *V. parahaemolyticus.* Little information is available on serotype genetic and antimicrobial profiles of *V. vulnificus* and *V. parahaemolyticus* recovered from Maryland estuaries. The aim of the present study was to determine the serotype of *V. parahaemolyticus*, evaluate antimicrobial susceptibility and genetic profiles of *V. vulnificus* and *V. parahaemolyticus* isolated from water and blue crab (*Callinectes sapidus*) samples collected from the Maryland Coastal Bays. One hundred and fifty (150) PCR confirmed *V. parahaemolyticus* including 52 *tdh*^+^ (pathogenic) and 129 *V. vulnificus* strains were tested for susceptibility to twenty (20) different antibiotics chosen by clinical usage for *Vibrio* species. The O serogroups were determined using an agglutination test with *V. parahaemolyticus* antisera. Pulsed-field gel electrophoresis (PFGE) was used for molecular subtyping to investigate the genetic diversity among tested strains. The most prevalent serotypes were O5 (33.3%), O3 (18.7%) and O1 (14.7%). More than 41% of all tested *Vibrio* isolates were resistant to three or more antibiotics. Cephalothin showed the highest resistance (42% and 61%), followed by cefoxitin (42% and 31%) and ceftazidime (36% and 29%) for *V. vulnificus* and *V. parahaemolyticus*, respectively. Most strains (99–100%) were susceptible to ampicillin/sulbactam, levofloxacin, piperacillin, piperacillin/tazobactam, and tetracycline. Fifty percent (50%) of the cephalothin resistant strains were crab isolates. *Vibrio vulnificus* and *V. parahaemolyticus* isolates demonstrated a high genetic diversity and 31% of *V. vulnificus* and 16% of *V. parahaemolyticus* strains were PFGE untypeable. No correlations were found between the *V. parahaemolyticus* serotype, pathogenicity, genetic and antimicrobial resistance profiles of both species of *Vibrio*. The observed high multiple drug resistance of *V. vulnificus* and *V. parahaemolyticus* from blue crab and its environment is of public health concern. Therefore, there is a need for frequent antibiotic sensitivity surveillance for *Vibrio* spp.

## Introduction

*Vibrio vulnificus* and *V. parahaemolyticus* are Gram negative non-spore forming rod-shaped halophilic bacteria naturally found in warm marine and estuarine environments (Oliver, 2013; Parveen and Tamplin, 2013). These bacteria are the leading cause of seafood-borne illness and mortality in the United States (Scallan et al., 2011). Persons with underlying medical conditions, such as alcoholism, liver disease, cancer, and diabetes may be at increased risk of infection and serious complications (Liu et al., 2006). *V. vulnificus* can cause septicemia and wound infection in immunocompromised individuals who eat contaminated seafood or have an open wound that is exposed to seawater (Oliver, 2013; Xu et al., 2014; Centers for Disease Control and Prevention (CDC), 2019).

*Vibrio* species produce many virulence factors including enterotoxins, hemolysins, cytotoxins, proteases, siderophores, adhesive factors, and hemagglutinins. *Vibrio parahaemolyticus* produces Thermostable Direct Hemolysin (TDH) and Thermostable Related Hemolysin (TRH) which are determinants of virulent strains (Broberg et al., 2011; Zhang and Orth, 2013; Letchumanan et al., 2014). There are two genetic markers for *V. vulnificus*: a species-specific marker- *V. vulnificus* hemolysin gene A (*vvhA*) and virulence correlated gene (*vcgC*) (Rosche et al., 2005).

Nationwide, approximately 80,000 cases of vibriosis including an average of 287 culture-confirmed cases of *V. parahaemolyticus* and 111 cases of *V. vulnificus*, 500 hospitalizations and 100 deaths of which 35 are *V. vulnificus* related deaths are reported every year (Scallan et al., 2011; Newton et al., 2012). The incidence of *Vibrio* infections has been increasing in the United States and there has been an increase in infections caused by a specific strain of *V. parahaemolyticus* due to climate change and rising water temperature. Before 2012, *V. parahaemolyticus* infections of this strain were rarely associated with shellfish from the United States Atlantic coast. Maryland is among the seven coastal states with increased incidence of vibriosis. In August 2012, a *V. parahaemolyticus* outbreak involving six persons occurred in Maryland and the outbreak isolates were linked to the O3:K6 pandemic clone of *V*. *parahaemolyticus* that have been observed throughout the world (Wang et al., 2017). In 2018, CDC reported a multistate outbreak of *V. parahaemolyticus* infections linked to eating fresh crab meat imported from Venezuela. Among 26 infected people, 15 (58%) were from Maryland [Centers for Disease Control and Prevention (CDC), 2018; Haendiges et al., 2014]. *V. vulnificus* is the leading cause of reported human death in the United States caused by the consumption of seafood. The increase of *V. vulnificus* infection is particularly concerning given the high mortality rate (35%) associated with the pathogen [Newton et al., 2012; Centers for Science in the Public Interest (CSPI), 2020]. For example, in 2016 a man died from *V. vulnificus* infection four days after cleaning crab pots in Ocean City, Maryland (Washington Post, 2016).

The recommended antibiotics for the treatment of *V. vulnificus* infections are doxycycline, third-generation cephalosporin (e.g., ceftazidime), fluoroquinolone (such as levofloxacin, ciprofloxacin or gatifloxacin), and for children, trimethoprim-sulfamethoxazole plus an aminoglycoside. The use of quinolones or tetracycline for treatments of *V. vulnificus* infections is associated with lower mortality than cephalosporin alone. Tetracycline or ciprofloxacin can also be used in severe or prolonged illnesses of *V. parahaemolyticus* (Wong et al., 2015; Centers for Disease Control and Prevention (CDC), 2019).

During the past few decades, many bacteria have acquired antimicrobial resistance due to the excessive use of antimicrobials in human, agriculture, and aquaculture systems (Park et al., 2018). Antibiotics are one of the “contaminants of emerging concern” that are increasingly occurring in livestock and poultry manure across the United States. According to the United States Environmental Protection Agency (EPA), manure accounts for 19% of nitrogen and 26% of phosphorous entering the Chesapeake Bay (Krikstan, 2013). Manure can contain antibiotics that could facilitate the development of antimicrobial resistance in bacteria. Marine bacteria exposed to antibiotics can develop antimicrobial resistance (Labella et al., 2013) transferable by mobile genetic elements and horizontal gene transfer and can cause changes in the coastal environment (Christaki et al., 2020). *Vibrio vulnificus* and *V. parahaemolyticus* are included in the natural microbial flora of the Chesapeake (DaSilva et al., 2012; Elmahdi et al., 2018; Parveen et al., 2020) and Maryland Coastal Bays, and have been isolated from crabs (*Callinectes sapidus*), oysters (*Crassostrea virginica*), water and sediment samples (Rodgers et al., 2014). Improperly cooked crabs and handling of live crabs represent a potential route of exposure to pathogenic and antimicrobial resistant strains of *V. vulnificus* and *V. parahaemolyticus*. The prevalence of *V. vulnificus* and *V. parahaemolyticus* in Maryland Coastal Bays is high, and the highest concentrations were found in crab compared to water and sediment (Rodgers et al., 2014). Despite the fact that the prevalence and ecology of *V. vulnificus* and *V. parahaemolyticus* is well documented in Maryland, especially in the Chesapeake Bay, little information is available on the serotype, and genetic and antimicrobial profiles of *V. vulnificus* and *V. parahaemolyticus* recovered from crab samples from the Maryland Coastal Bays. The aim of the present study was to determine the phenotypic and genotypic characteristics of *V. vulnificus* and *V. parahaemolyticus* strains isolated from crab, seawater and sediment samples collected from the Maryland Coastal Bays.

## Materials and Methods

### Bacterial Strains and Sample Collection

**FIGURE 1:**
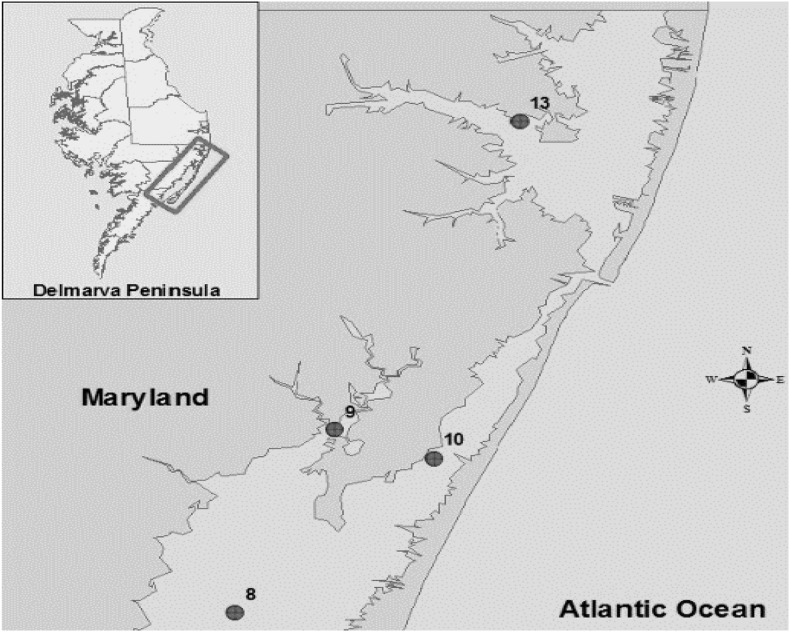
Location of sampling sites in the Maryland Coastal Bays. 8- Chincoteague Bay, 9- Newport Bay, 10- Sinepuxent Bay, 13–St. Martin River. Courtesy of Tracie J. Bishop and Andres G. Morales-Nunez (Rodgers et al., 2014).

The bacterial strains were isolated previously (Rodgers et al., 2014) from crab hemolymph, sediment and water samples collected from the Maryland Coastal Bays including Chincoteague Bay (site 8), Newport Bay (site 9), Sinepuxent Bay (site 10), and St. Martin River (site 13) (Figure 1). *V. parahaemolyticus* and *V. vulnificus* strains were stored at −80°C in the cryogenic vials for further analysis.

### Serotyping

The identification of somatic (O) serotype of *V. parahaemolyticus* strains was performed with 11 commercially available antisera using slide agglutination test according to the manufacturer’s recommendations (Denka Seiken Co., Ltd., Tokyo, Japan).

### Antimicrobial Susceptibility Testing

*Vibrio* isolates were tested for susceptibility to antibacterial drugs using the microbroth dilution method according to the guidelines of the Clinical and Laboratory Standards Institute (CLSI). For this testing, twenty different antibiotics were chosen based on clinical usage for *V. vulnificus* and *V. parahaemolyticus*. There were amikacin (AMI; 4–64 μ/ml), amoxicillin/clavulanic acid (AMC; 2/1–16/8 μ/ml), ampicillin (AMP; 2–16 μ/ml), ampicillin/sulbactam (SAM; 2/1–16/8 μ/ml), cefepime (FEP; 0.5–16 μ/ml), cefotaxime (FOT; 0.03–2 μ/ml), cefoxitin (FOX; 4–32 μ/ml), ceftazidime (TAZ; 4–32), ceftriaxone (AXO; 0.5–16 μ/ml), cephalothin (CEP; 2–16 μ/ml), ciprofloxacin (CIP; 0.25–2 μ/ml), chloramphenicol (CHL; 2–16 μ/ml), doxycycline (DOX; 0.5–8 μ/ml), imipenem (IMI; 1–8 μ/ml), levofloxacin (LEVO; 0.5–4 μ/ml), meropenem (MERO; 0.25–8 μ/ml), piperacillin (PIP; 1–64 μ/ml), piperacillin/tazobactam (P/T4; 1/4–32/4 μ/ml), tetracycline (TET; 0.5–8 μ/ml), and trimethoprim/sulfamethoxazole (SXT; 2/38–4/76 μ/ml). The Minimal Inhibitory Concentration (MIC) was recorded as the lowest concentration of antimicrobial agent with no visible growth. Multidrug resistance was defined as an absence of susceptibility to two or more classes of antibiotics. *Escherichia coli* ATCCH25922 and *Staphylococcus aureus* ATCCH29213 were used as controls [Clinical and Laboratory Standards Institute (CLSI), 2010; Elmahdi et al., 2018].

### Pulsed Field Gel Electrophoresis

Pulsed-field gel electrophoresis (PFGE) was used to investigate the genetic variation of the *Vibrio* isolates with different antibiotic susceptibility profiles. Plug preparation, digestion and separation of DNA fragment and PFGE were performed using the *Sfi*I restriction enzyme as described in the CDC Pulse-Net protocol for *V. parahaemolyticus* and *V. vulnificus* (PulseNet, United States 2013; Parsons et al., 2007; Kam et al., 2008; Elmahdi et al., 2018). To improve the typeability of some strains 50 μM of thiourea was added to electrophoresis buffer [0.5X TBE (*Tris*-Borate EDTA)] (Banerjee and Farber, 2009). The gel was stained with ethidium bromide, and DNA bands were visualized with a UV transilluminator. Cluster analysis of PFGE was performed using bionumerics software (version 7.6, Applied Maths, Austin, TX, United States). Dice coefficient was calculated according to unweighted-pair group method with arithmetic mean. Isolates were assigned to the same macrorestriction profile when they clustered within > 99% pattern similarity (1.2% optimization and 1.5% position tolerance).

### Statistical Analysis

One-Way Analysis of Variance (ANOVA) and Pearson’s Chi square test were used to analyze the data and compare the means among sites, sample type, antibiotic resistance patterns of *V. vulnificus* and *V. parahaemolyticus* isolates, and to evaluate the differences in the occurrence of antimicrobial susceptibility and correlation between the sample sites, types pathogenicity, Genetic and antimicrobial resistance profiles using SPSS version 26.0 (IBM Corp., NY, United States).

## Results

### Serotype

Serotype was performed in all 150 *V. parahaemolyticus* strains. Except for O7 and O9, all serogroups were equally distributed in all four sites in Maryland Coastal Bays (Table 1). The most prevalent serotypes were O5 (33.3%), O3 (18.7%), and O1 (14.7%). Serotypes O2 and O8 were rarely found (Table 1). Twenty-seven (52%) of O5 strains were multidrug resistant and 19 (37%) of these strains were pathogenic (18 *tdh*^+^ and 1 *tdh*^+^ and *trh*^+^). However, there was no correlation between serotype and pathogenicity (*r* = 0.36, *p* > 0.05).

**TABLE 1:** Distribution of *Vibrio parahaemolyticus* O serogroups in four sites of Maryland Coastal Bays.

**Sero group**	**Chincoteague Bay**	**Newport Bay**	**Sinepuxent Bay**	**St. Martin River**	**Total**
O1	4	8	7	3	22
O2	1	0	0	1	2
O3	8	6	6	8	28
O4	3	1	3	4	11
O5	13	12	17	8	50
O6	2	4	1	5	12
O7	0	0	0	0	0
O8	1	0	2	0	3
O9	0	0	0	0	0
O10	3	3	3	0	9
O11	0	5	3	5	13

### Antimicrobial Profile

**FIGURE 2:**
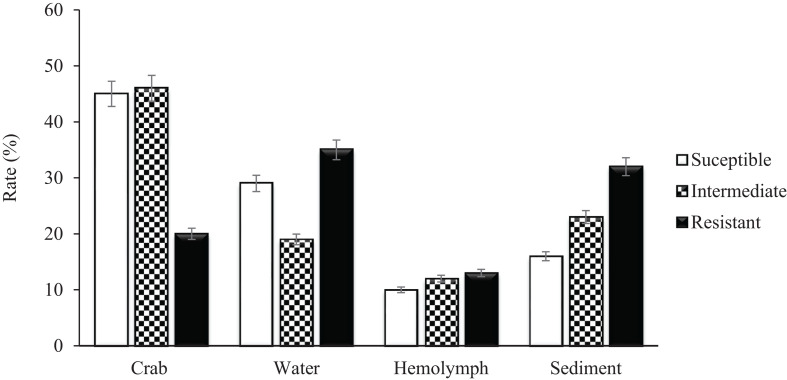
Percentage of *Vibrio vulnificus* isolates susceptible, intermediate and resistant to Cephalothin.

**FIGURE 3:**
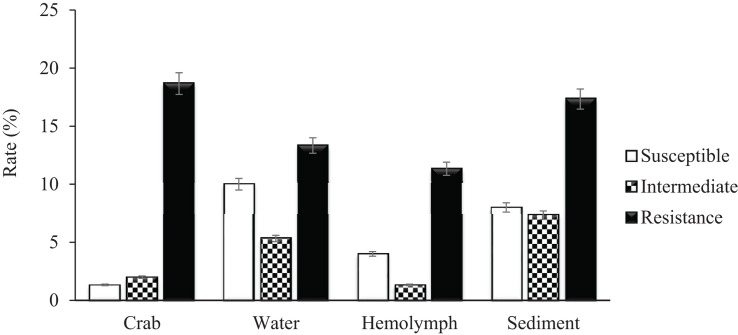
Percentage of *Vibrio parahaemolyticus* susceptible, intermediate and resistant to cephalothin.

A total of 279 (129 *V. vulnificus* and 150 *V. parahaemolyticus)* strains were tested for antibiotic sensitivity. Sixty-five percent (65%) of all tested *V. vulnificus* and *V. parahaemolyticus* strains were resistant to one or more classes of antibiotics and 42% were multidrug resistant. The highest resistance was observed for cephalothin, 42% for *V. vulnificus* and 61% for *V. parahaemolyticus*, followed by cefoxitin (42 and 31%), ceftazidime (36 and 29%), ceftriaxone (35 and 30%), cefotaxime (27 and 20%), cefepime (21 and 20%), amikacin (20%) and meropenem (16 and 19%) for *V. vulnificus* and *V. parahaemolyticus*, respectively. Ninety-five to 100% of both *Vibrio* species were susceptible to most of the commonly used antibiotics such as ampicillin, ampicillin/sulbactam, doxycycline, levofloxacin, piperacillin, piperacillin/tazobactam, tetracycline and trimethoprim/sulfamethoxazole (Table 2). Isolates recovered from water and sediment had significantly (*p* ≤ 0.05) higher cephalothin resistance than those from crab meat and hemolymph (Figures 2, 3).

**TABLE 2:** *Vibrio vulnificus* and *Vibrio parahaemolyticus* antimicrobial profile.

**Antibiotic**	**Conc. μ/ml**	**Susceptible (%)**		**Intermediate (%)**		**Resistance (%)**	
		***Vv***	***Vp***	***Vv***	***Vp***	***Vv***	***Vp***
Amikacin**	4–64	74	75	5	5	20	20
Amoxicillin/Clavulanic acid	2/1–16/8	89	65	7	18	4	17
Ampicillin	2–16	96	95	3	4	1	1
Ampicillin/Sulbactam	2/1–16/8	100	100	0	0	0	0
Cefepime	0.5–16	67	68	12	8	21	24
Cefotaxime**	0.03–2	66	75	7	4	27	21
Cefoxitin	4–32	30	60	29	9	42	31
Ceftazidime	4–32	57	69	6	2	36	29
Ceftriaxone**	0.5–16	64	65	2	5	35	30
Cephalothin	2–16	38	23	20	16	42	61
Chloramphenicol	2–16	98	98	1	1	1	1
Ciprofloxacin**	0.25–2	98	95	2	5	0	0
Doxycycline**	0.5–8	100	97	0	1	0	1
Imipenem	1–8	84	65	13	17	3	17
Levofloxacin**	0.5–4	100	99	0	0	0	1
Meropenem	0.25–8	74	73	11	9	16	19
Piperacillin	1–64	99	99	1	0	0	1
Piperacillin/Tazobactam	1/4–32/4	100	99	0	0	0	1
Tetracycline**	0.5–8	100	99	0	1	0	1
Trimethoprim/Sulfamethoxazole	2/38–4/76	100	97	0	0	0	3

Vv, Vibrio vulnificus; Vp, Vibrio parahaemolyticus. **CDC recommended antibiotics for Vibrio infection treatment.

### Pulsed Field Gel Electrophoresis

**FIGURE 4:**
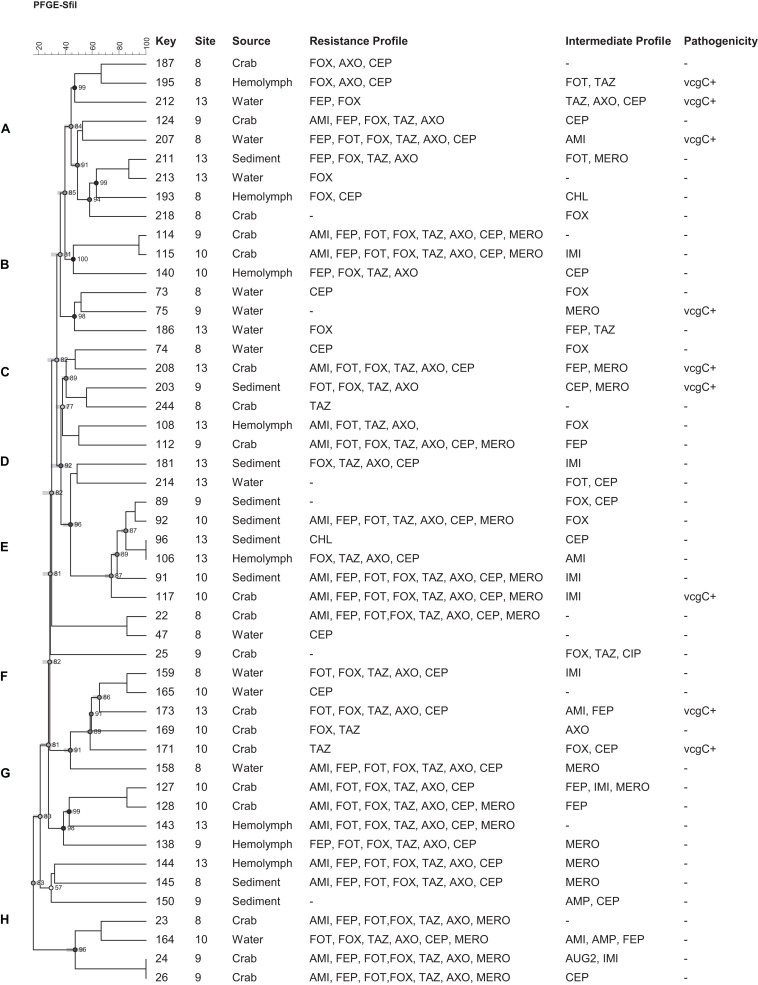
Dendrogram of PFGE profiles of *Vibrio vulnificus* strains collected form crab and its environment in Maryland Coastal Bays; Site 8- Chincoteague Bay, 9- Newport Bay, 10- Sinepuxent Bay, 13–St. Martin River. Key = stain number. AMI, amikacin; AUG2, amoxicillin/clavulanic acid; AMP, ampicillin; A/S2 ampicillin-sulbactam; FEP, cefepime; FOT, cefotaxime; FOX, cefoxitin; TAZ, ceftazidime; AXO, ceftriaxone; CEP, cephalothin; CIP, ciprofloxacin; CHL, chloramphenicol; DOX, doxycycline; IMI, imipenem; LEVO, levofloxacin; MERO, meropenem; PIP, piperacillin; P/T4, piperacillin/tazobactam; TET, tetracycline; SXT, trimethoprim/sulfamethoxazole. *vcgC*, virulence-correlating gene type C. Letters A to H on the left represent pulsed-field gel electrophoresis clusters; key represent strain numbers. Minus sign on the resistance and intermediate profiles columns indicate “no resistance” or “no intermediate” profiles and on the Pathogenicity column (-) mean vcgC gene negative.

**FIGURE 5:**
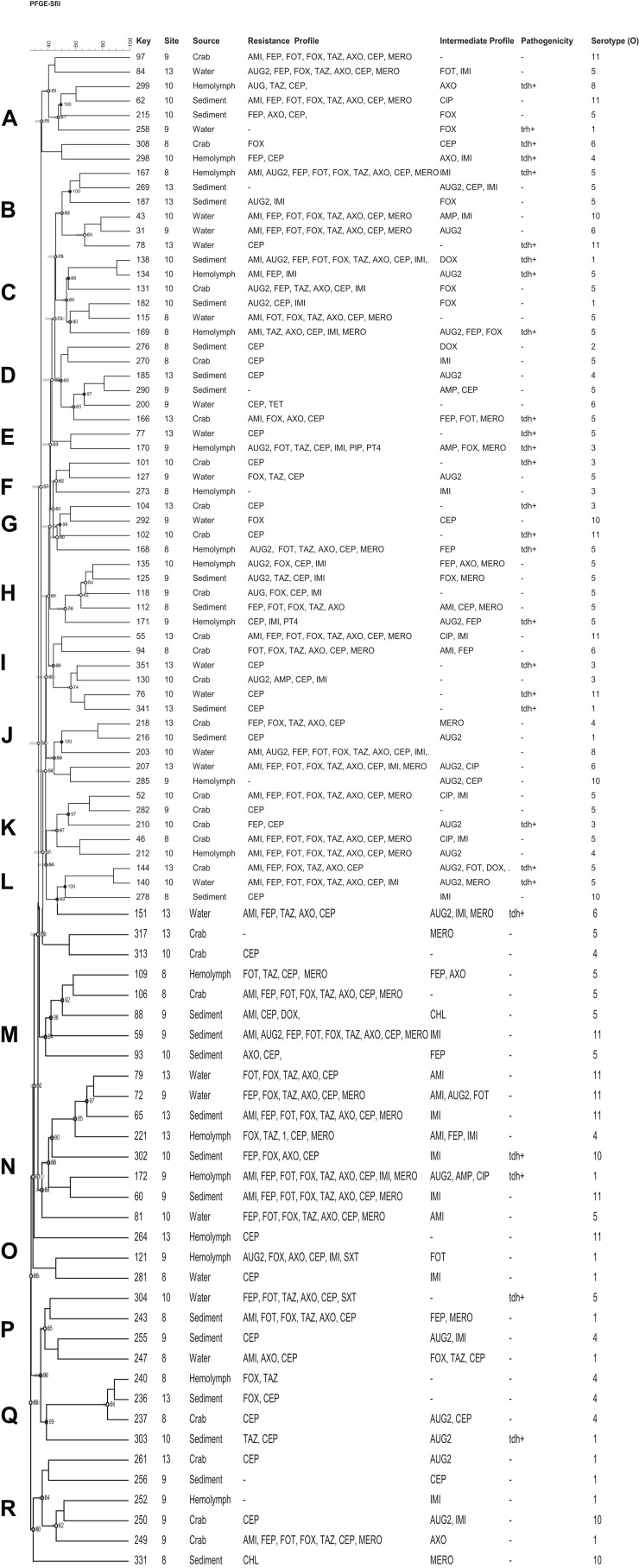
Dendrogram of PFGE profiles of *Vibrio parahaemolyticus* strains collected form crab and its environment in Maryland Coastal Bays; Site 8–Chincoteague Bay; 9–Newport Bay; 10–Sinepuxent Bay; 13–St. Martin River. Key = stain number. AMI, amikacin; AUG2, amoxicillin/clavulanic acid; AMP, ampicillin; A/S2, ampicillin-sulbactam; FEP, cefepime; FOT, cefotaxime; FOX, cefoxitin; TAZ, ceftazidime; AXO, ceftriaxone; CEP, cephalothin; CIP, ciprofloxacin; CHL, chloramphenicol; DOX, doxycycline; IMI, imipenem; LEVO, levofloxacin; MERO, meropenem; PIP, piperacillin; P/T4, piperacillin/tazobactam; TET, tetracycline; SXT, trimethoprim/sulfamethoxazole; tdh, thermostable direct hemolysin. Letters A to R on the left represent pulsed-field gel electrophoresis clusters; key represent strain numbers; minus sign (-) on the resistance and intermediate profiles columns indicates “no resistance” or “no intermediate” profiles and on the Pathogenicity column (-) indicates *tdh* and *trh* negative; Serotype (O) = +O1–O11.

Based on the antimicrobial resistance profiles and typeability, 49 (including 9 *vcg*C^+^) *V. vulnificus* and 92 (including 28 *tdh*^+^) *V. parahaemolyticus* strains from different sources were selected and a dendrogram was constructed based on *Sfi*I PFGE patterns to compare the banding profiles of these bacteria. Thirty-one percent (31%) of *V. vulnificus* and 16% of *V. parahaemolyticus* strains were PFGE untypeable and failed to yield discernible patterns. Both vibrio species demonstrated a high genetic diversity and they were grouped into 8 (A to H) *V. vulnificus* and 19 (A to R) *V. parahaemolyticus* clusters of related patterns of ≥ 75% similarity. No relationship was observed between the genetic and antimicrobial resistance profiles of both *Vibrio* species. Five (5) *V. vulnificus* (number 91, 117, 114, 115, and 22) and 10 *V. parahaemolyticus* (number 97, 62, 43, 31, 55, 52, 210, 212, 106, and 65) strains showed similar antimicrobial resistance profile, although they were genetically different (Figures 4, 5).

## Discussion

For epidemiological perspective, *V. parahaemolyticus* strains were serotyped. The most prevalent serotypes were O5, O3 group that have been observed around the world, and O1; followed by O6 and O4. Serotypes O2 and O8 were very scarce; whereas O7 and O9 were not found in the Maryland Coastal Bays (Table 1). Similar results were found in the Chesapeake Bay oysters and their environment, where the most isolated groups were O3, O1, O5, and O7, and O9 were not found (de Hernández-Díaz et al., 2015; Chen et al., 2017; Elmahdi et al., 2018). In the present study we did not determine the K type of our strains. In 2009, Chao and his collaborators isolated other serotypes of the pandemic strain (O1:K36, O3:K25, and O3:K68), in China and in 2009 also reported another serotype (O3:K5) in the American continent (Chao et al., 2009; Velazquez-Roman et al., 2014). A recent study conducted by Siddique et al. (2021) on *V. parahaemolyticus* isolated from fish aquaculture in Bangladesh, the majority of strains contain O8 antigen followed by O5, O11, O3, and O1. Most of the strains could not be typed serologically for K (KUT) antigen using conventional kits. Similar to our results they did not report O7 and O9 serotypes. Besides of O3 group, O4:K12 and O4: K (unknown) are pandemic *V. parahaemolyticus* strains of the Pacific Northwest associated with outbreaks in New York, Atlantic Coast of Spain in 2012 and in 13 United States Atlantic Coast states in 2013 (Centers for Disease Control and Prevention (CDC), 2013; Newton et al., 2014). It is highly recommended that future studies perform K-typing to identify and enumerate the pandemic serotype in the Maryland Coastal Bays.

*Vibrio vulnificus* and *V. parahaemolyticus* strains exhibited similar antimicrobial profiles and contain high percentage of multidrug resistant strains that were distributed along the four sites (Chincoteague Bay, Newport Bay, Sinepuxent Bay and St. Martin River) (*p* > 0.0%) in the Maryland Coastal Bays. Both *Vibrio* species showed the highest resistance to cephalothins, one of the recommended antibiotics for vibriosis treatment, followed by cefoxitin, ceftazidime, ceftriaxone, cefotaxime, cefepime, amikacin and meropenem. *V. parahaemolyticus* strains showed higher resistance to cephalothin compared to *V. vulnificus*. Resistant to commonly used antibiotic is also elevated in *V. parahaemolyticus* than *V. vulnificus* (Table 2). All tested *Vibrio* strains (100%) were susceptible to ampicillin/sulbactam, and all *V. vulnificus* strains were susceptible to levofloxacin, piperacillin, piperacillin/tazobactam, tetracycline and trimethoprim/sulfamethoxazole (Table 2). Half of the cephalothin resistant strains were crab isolates. Water and sediment samples had significantly (*p* ≤ 0.05) higher cephalothin resistant strains compared to crab meat and hemolymph samples (Figures 2, 3); there was no significant difference among the sites. No correlations were found between the serotype, pathogenicity and antimicrobial resistance profiles. Similar antimicrobial profiles for both, *V. vulnificus* and *V. parahaemolyticus* were found by Elmahdi et al. (2018) and Shaw et al. (2014). However, Shaw’s team found higher susceptibility of *V. vulnificus* (95%) and *V. parahaemolyticus* (82%) to cephalothin versus 38 and 23% for *V. vulnificus* and *V. parahaemolyticus*, respectively observed in the present study.

It has been reported that molecular typing is a reliable and useful tool for investigating the genetic diversity and tracking sources of contamination of food and waterborne pathogens in aquatic systems and food processing plants (Mohamed et al., 2014; Elmahdi et al., 2018). In this study, dendrogram of PFGE cluster analysis of 141 *V. vulnificus* and *V. parahaemolyticus* strains from crabs and its environments (water, sediment, crab meat and hemolymph) were performed according to the standard PulseNet PFGE protocol for *Vibrio* spp. with the restriction enzyme *Sfi*I. Though a few *V. vulnificus* and *V. parahaemolyticus* strains recovered from seafood in the United States, specifically in Maryland have been genetically characterized by PFGE analysis, this is the first study that reported the PGFE profiles of *V. vulnificus* and *V. parahaemolyticus* strains recovered from crabs and its surrounding environments in the Maryland Coastal Bays.

In this study, eight clusters (Figure 4; marked with letter A to H on the left) for *V. vulnificus* were found with different 80% similarity in banding patterns. Multidrug resistant as well as potential pathogenic strains of both species of *Vibrio* were distributed in different clusters. Only two strains out of 49 *V. vulnificus* strains fell into the same cluster, with 100% similarity and exhibited the same multidrug resistant pattern. Moreover, these isolates were from the same site (site 9) and source (crab); however, they differed in intermediate antibiotic profile (strain #24 showed intermediate profile for AUG2 and IMI, whereas strain # 26 showed intermediate profile for CEP). *V. vulnificus* showed higher percentage (≥80%) of similarity compare to *V. parahaemolyticus* (≥75%); however, these differences were not statistically significant (*p* > 0.05). The PFGE profiles of *V. parahaemolyticus* (Figure 5) were genetically diverse, and no genetic relationship was found among and between the sampling sites, antimicrobial profile, pathogenicity, and serogroups. All recovered *V. parahaemolyticus* isolates clustered (A to R) at 75% or higher similarity in banding patterns. These results indicate high intraspecific diversity of this species. Our findings are consistent with a previous study that reported high genetic heterogeneity among *V. vulnificus* and *V. parahaemolyticus* isolates recovered from oysters and water during a relaying study in the Chesapeake Bay, Maryland (Elmahdi et al., 2018). Moreover Chen et al. (2017) observed a high genetic diversity among *V. vulnificus* and *V. parahaemolyticus* isolates recovered from oysters and water collected from the Chesapeake Bay, Maryland. The percentage of untypeable stains of *V. vulnificus* (31%) was higher than *V. parahaemolyticus* (16%) despite the fact that 50 μM of thiourea was added to 0.5X TBE (Tris-Borate EDTA) buffer for the repeated PFGE experiments. Only 4 *V. parahaemolyticus* and 1 of *V. vulnificus* strains were typeable after the addition of thiourea in the electrophoresis buffer. Fawley and Wilcox (2002) found that of 200 μM of thiourea must be present in both agarose gel and the electrophoresis buffer to ensure minimal DNA degradation. High level of untypeable *V. vulnificus* strain was also reported in studies of PFGE analysis of *V. vulnificus* strains isolated from Taiwan and the United States (Wong et al., 2004). Therefore, it is important to analyze these untypeable isolates using a more sensitive and specific method, whole genome sequencing to reveal the reason for the untypeability.

## Conclusion

The PFGE profiles of *V. vulnificus and V. parahaemolyticus* stains isolated from Maryland Coastal Bays were diverse. No genetic relationship was found among the sampling sites, antimicrobial resistance profile, and pathogenicity. The observed high multiple drug resistance of *V. vulnificus* and *V. parahaemolyticus* from blue crab and its environment is of public health concern, despite the fact that these bacteria were susceptible to the two CDC recommended antibiotics (Tetracycline 99–100% and Ciprofloxacin 95–98%) for its treatment. Therefore, frequent antibiotic sensitivity surveillance is needed.

## Data Availability Statement

The raw data supporting the conclusions of this article will be made available by the authors, without reservation.

## Author Contributions

SP, PC and LS contributed to the conception and design of the study. LS and SO were involved in experimental procedures in the laboratory and data collection and analysis. The manuscript was written by LS, and reviewed, edited, and approved by all authors. SP and PC were responsible for the integrity of the work and overall supervision.

## Conflict of Interest

The authors declare that the research was conducted in the absence of any commercial or financial relationships that could be taken as a potential conflict of interest.
